# Moyamoya disease with distal anterior choroidal artery aneurysm resected via transcallosal approach: A case report and review

**DOI:** 10.1097/MD.0000000000033973

**Published:** 2023-06-02

**Authors:** Wei Ding, Yunfei Zhao, Lei Liu, Peng Wang, Wenchuan Qiu, Hongwei Ren, Shengxi Jin

**Affiliations:** a Department of Neurosurgery, Tianyou Hospital of Wuhan University of Science and Technology, Wuhan, China; b Department of Medical Imaging, Tianyou Hospital of Wuhan University of Science and Technology, Wuhan, China.

**Keywords:** aneurysm, anterior choroidal artery, craniotomy, intraventricular hemorrhag, moyamoya disease, third ventricular

## Abstract

Moyamoya disease (MMD) is a cerebrovascular structural disorder characterized by bilateral stenosis and obstruction of the internal carotid artery, anterior cerebral artery, and initial segment of a middle cerebral artery, as well as the aberrant formation of collateral arteries at the base of the brain. Moyamoya disease with distal anterior choroidal artery (AChA) aneurysm is extremely uncommon. At present, the treatment of Moyamoya disease with aneurysm mainly includes conservative treatment and surgical treatment, including revascularization, endovascular therapy and microsurgical clipping or resection. Interventional therapy is the first treatment of choice. For those whose paths are tortuous and inaccessible and intervention fails, I successfully excised them through craniotomy.

**Patient concerns:**

The 38-year-old male patient, diagnosed with Moyamoya disease 11 years ago and was hospitalized for multiple intraventricular hemorrhages throughout that time. During the 11 years, the patient was hospitalized for intra ventricular hemorrhage for several times. The patient was diagnosed as moyamoya disease for many times by digital subtraction angiography, but he was recommended to come to our hospital for cerebrovascular bypass surgery 3 months after each hemorrhage, but he did not come to our hospital until the next intraventricular hemorrhages.

**Diagnoses:**

This recurrent intraventricular bleeding was suspected to be caused by MMD, and a digital subtraction angiography of the brain revealed an aneurysm of the distal AChA.

**Interventions:**

Interventional therapy was the first choice. During the operation, transcatheter aneurysm embolization was tried. Finally, interventional therapy was abandoned because the vessels were too thin and tortuous and the guide wire could not pass through. After detecting the aneurysm using computerized tomography angiography, the distal AChA aneurysm was resected through the lateral interventricular foramen of the corpus callosum, and the corpus callosum was parted along the interhemispheric fissure to access the third ventricle.

**Outcomes:**

On the 21st postoperative day, the patient improved, recovered to a Glasgow Coma Scale score of 15.

**Lessons:**

We conclude that craniotomy is a satisfying alternative in patients with MMD complicated by perforated distal AChA aneurysm hemorrhage if the vascular prerequisites for endovascular treatment are not accessible and the patient has a favorable prognosis.

## 1. Introduction

Japanese researchers Takeuchi and Shimizu first reported moyamoya disease (MMD) in 1957.^[1]^ MMD is defined by persistent progressive stenosis and obstruction of the internal carotid artery and its distal arteries on both sides of the head, as well as anomalies of the tiny reticular vessels near the base of the brain. It can be ischemic, bleeding, or asymptomatic.^[2]^ The prevalence of aneurysms in MMD patients has been reported to be substantially greater than in the general population, ranging from 3.4% to 14.8%. According to current studies, aneurysm formation in persons with moyamoya illness is connected to genetic, immunological, blood flow, and vascular factors.^[3]^ According to the site of the aneurysm, moyamoya disease complicated by intracranial aneurysm is divided into the aortic aneurysm and peripheral aneurysm. Aortic aneurysm rupture frequently results in subarachnoid hemorrhage. Patients experience severe headaches as a result of this. Peripheral aneurysms generate intracerebral sulcus and intraventricular bleeding, most frequently in the basal ganglia, thalamus, and subcortical areas. Computerized tomography(CT) examination has a high diagnostic value for cerebral hemorrhage caused by aneurysm rupture in moyamoya disease, but it has a low diagnostic value for vascular morphology and proliferation. These disadvantages can be overcome with brain computerized tomography angiography (CTA) imaging. CTA can depict the location of stenotic vessels and aneurysms, and their 3-dimensional spatial relationship with adjacent tissue, which are crucial information to facilitate the surgical procedure.^[4]^ Because of signal attenuation at regions of hemodynamic complicated vasculature, magnetic resonance angiography has limited diagnostic use for moyamoya disease with associated aneurysms. For aneurysms with diameter < 3mm, the detection rate of digital subtraction angiography (DSA) was higher than that of CTA. The management of MMD with aneurysm includes indirect operation and direct operation. If the aneurysm is not ruptured, you can choose cerebral revascularization. After revascularization, some aneurysms become small or disappear on their own, an early bypass usually resulted in rapid neurological improvement.^[5,6]^ The key to the treatment of ruptured aneurysms is to block or remove the aneurysms. Endovascular embolization and microsurgical clamping or resection can achieve the above purpose.^[7]^ We describe an apathetic male patient with MMD who found an aneurysm in the distal anterior choroidal artery (AchA) and successfully resected the aneurysm after craniotomy with remarkable results.

## 2. Case report

**Figure 1.**
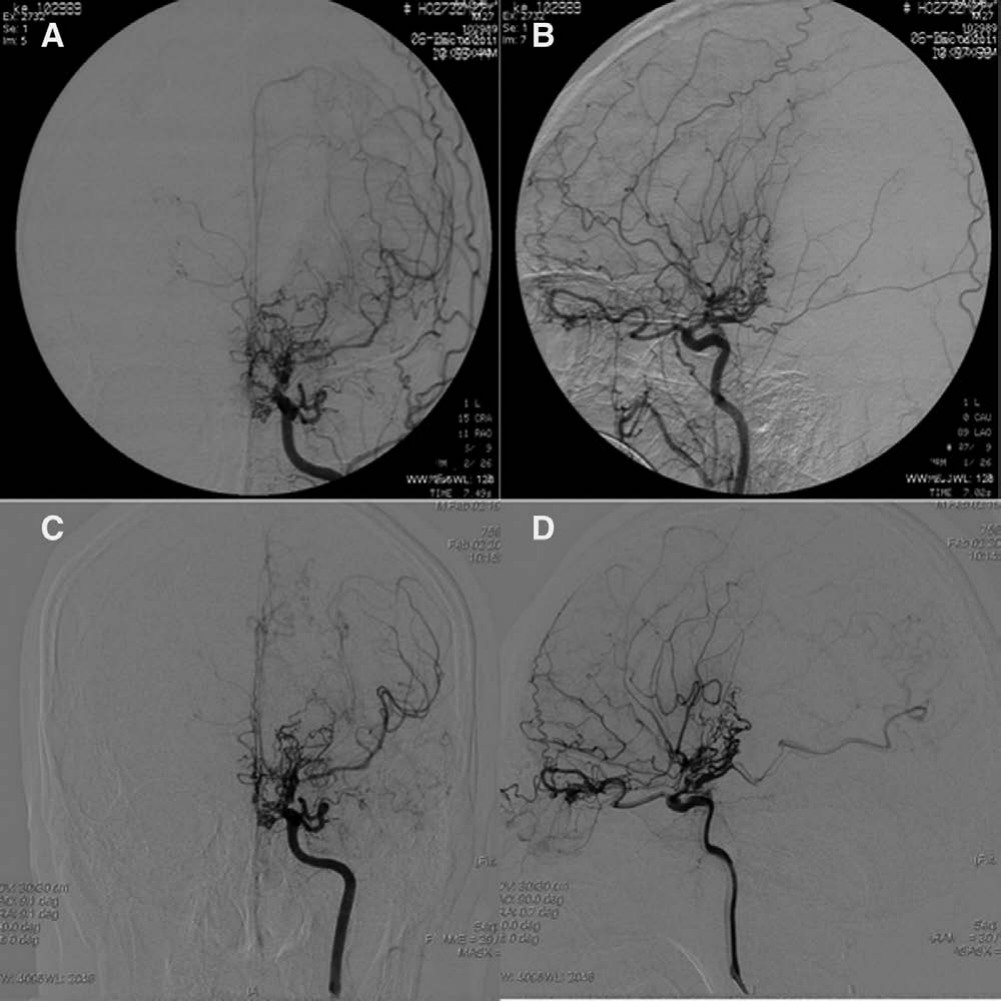
The DSA images of patints in 2011 and 2018. (A,B) shows brain DSA images from the patient’s hospitalization in 2011, disordered collateral vessels can be seen. (C,D) are images of the cerebral vasculature taken during the DSA examination on the brain in 2018, compensatory hyperplasia of blood vessels. DSA = digital subtraction angiography.

The patient, a 38-year-old male with a history of hypertension dating back 11 years, was admitted to the hospital for dizziness. In April 2011, the patient suddenly went unconscious, vomited, and experienced jet lag-like symptoms. His condition did not improve after treatment at a nearby hospital. The patient’s family referred him to the department of neurosurgery at Tianyou Hospital of Wuhan University of Science and Technology. The patient was admitted in a coma with a glasgow coma scale (GCS) score of 7 (E1V1M5). A physical examination indicated a 3-finger stiff neck, and a CT scan of the brain revealed intraventricular bleeding in the arachnoid. In the context of intraventricular hemorrhage, we conducted an emergency intracerebroventricular hematoma excision and tracheotomy on the patient. After 1 week of treatment, the patient progressively became awake, his GCS improved to 14 points, and his neck stiffness alleviated. DSA brain imaging and angiography of the bilateral internal carotid and vertebral arteries revealed uneven stenosis and truncation of the terminal segments of the bilateral internal carotid artery, the middle cerebral artery, and the anterior cerebral artery. The stenotic artery has several distal branches, all of which are darkened, distorted, and disordered arteries for collateral circulation. The right posterior cervical communicating artery and the posterior cerebral artery were both thickened and tortuous. The basilar artery segment of the left vertebral artery, which was involved in arterial circulation in the left cerebral blood supply area, was thickened, as demonstrated in the angiography (Fig. 1 A and B). The patient was diagnosed with MMD (Suzuki stage 3). After thorough preparation, we treated MMD aggressively, performed right superficial temporal artery and right temporalis muscle dressings. After a series of treatments, the patient’s GCS score was restored to 15 points.

In March 2015, the patient suffered headache symptoms and was transported to the department of neurosurgery at Tianyou Hospital of Wuhan University of Science and Technology, where he was diagnosed with moyamoya disease-related intraventricular hemorrhage and underwent external ventricular drainage. After treatment, the intraventricular hemorrhage was completely eradicated with remarkable efficiency. We suggested that patients should undergo right superficial temporal artery to middle cerebral artery (superficial temporal artery-middle cerebral artery) bypass surgery and brain DSA, but it was rejected by the patient.

In March 2016, the patient returned to the department of neurosurgery at Tianyou Hospital of Wuhan University of Science and Technology with dizziness and headache once more. The patient experienced around 3 fingers of neck stiffness at the time, and a CT scan of the head revealed ventricular system bleeding, subarachnoid hemorrhage. We did extra ventricular drainage once more on the day of admission. The patient was advised to undergo brain DSA, which was rejected again for economic reasons. He was discharged from the hospital.

In February 2018, the patient was admitted to the department of neurosurgery at Tianyou Hospital of Wuhan University of Science and Technology with symptoms of impaired consciousness and vomiting. A head CT revealed intraventricular hemorrhage and hydrocephalus as a result of MMD progression. The patient’s family refused extra ventricular drainage. reexamination of the brain DSA shows that the middle and anterior cerebral arteries terminal segments were blocked, and the bilateral internal carotid arteries were severely stenosed (approximately 50 percent stenosis at the narrowest spot). A high number of collateral circulation vascular shadows could be visible distal to the blocked artery. Angiography of the left vertebral artery revealed thickening of the basilar artery segment and significant thickening of the left posterior cerebral artery, both of which were involved in arterial circulation in the left cerebral blood supply area (Suzuki stage 3). (Fig. 1 C and D). After conservative treatment, such as prevented rebleeding, controlled blood pressure, prevented stress ulcer and so on. Then, the patient was discharged.

**Figure 2.**
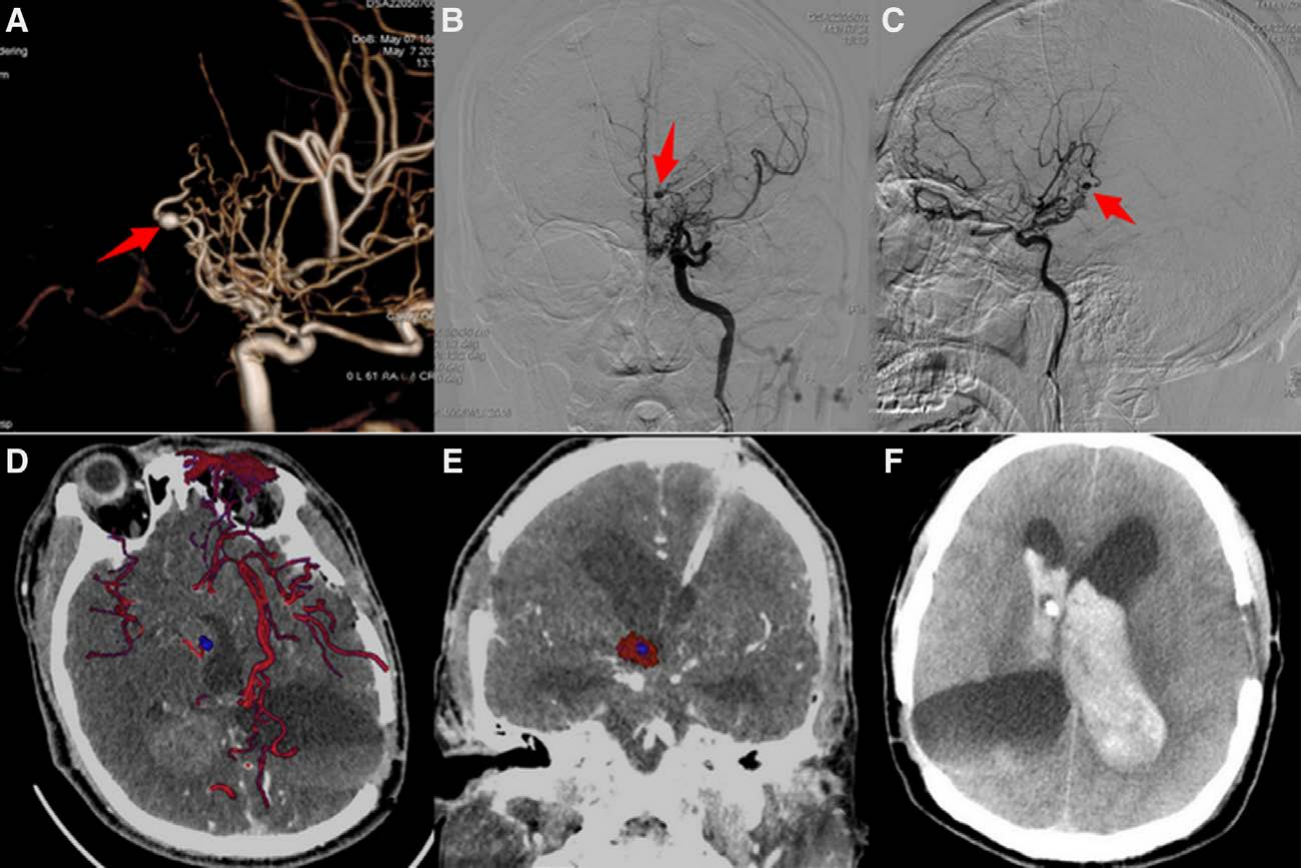
Preoperative imaging results of the patient. (A,B, and C) are images taken during the brain DSA examination while the patient was hospitalized and clearly show the 3-dimensional interaction between the aneurysm and the cerebral arteries (red arrows mark the aneurysm). (D, E) are available images before the operation. Multimodal CTA images clearly show the relationship between aneurysm, ventricle, and hematoma (red partarrows mark the hematoma, blue partarrows mark the aneurysm). (F) is the CT slice showing increased intraventricular hemorrhage before surgery. CT = computerized tomography, CTA = computerized tomography angiography, DSA = digital subtraction angiography.

**Figure 3.**
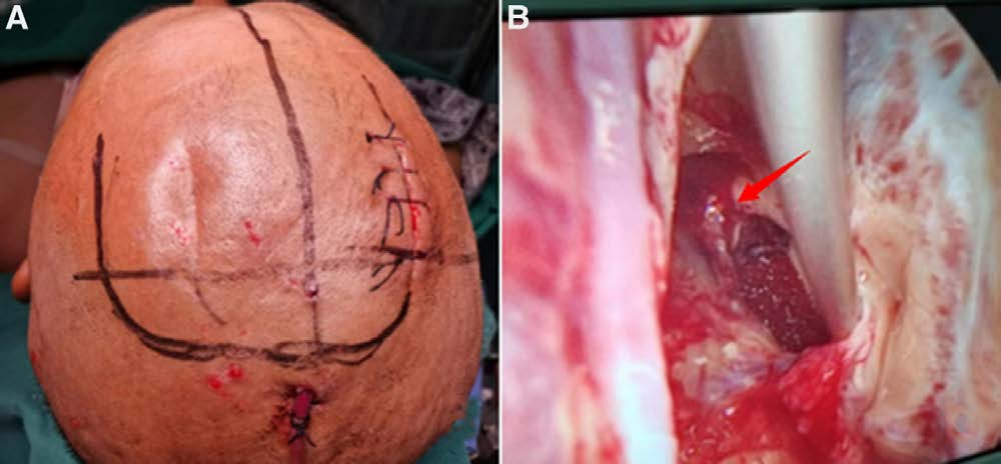
Intraoperative photograph of the patient. (A) shows the preoperative surgical incision designed for this patient. (B) is a photograph taken during the removal of the aneurysm through the interventricular foramen of the lateral corpus callosum, which clearly shows the aneurysm in the third ventricle (red arrows mark the aneurysm).

**Figure 4.**
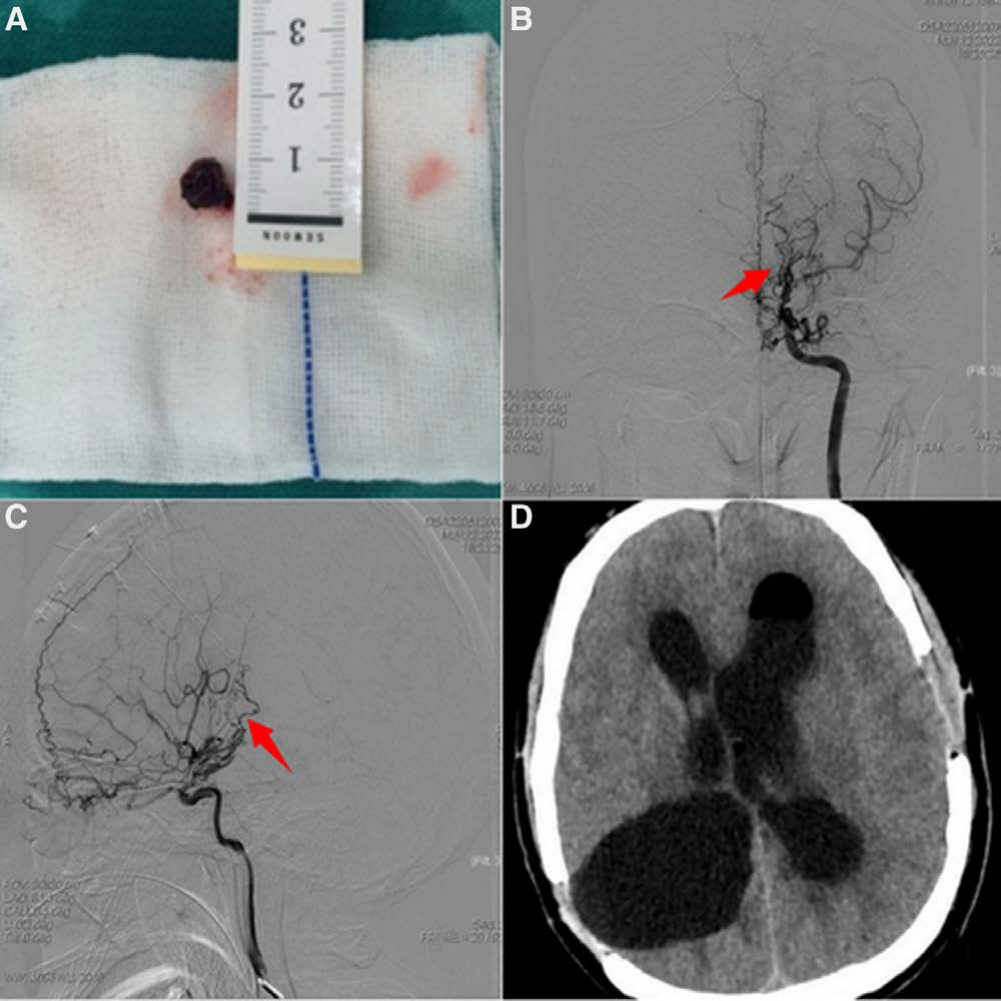
Postoperative imaging results of the patient. (A) is the aneurysm removed by craniotomy. (B,C) are cerebral vascular images taken during DSA of the patient’s brain after craniotomy, showing that the aneurysm has dissipated (red arrows mark the area of prior aneurysm), the anterior choroidal artery opacifies normally. (D) is a CT of the head taken before the patient was discharged, demonstrating the complete disappearance of the intracerebroventricular hematoma. CT = computerized tomography, DSA = digital subtraction angiography.

On April 30, 2022, the patient was referred to the Department of Neurosurgery at Tian You Hospital of Wuhan University of Science and Technology after experiencing headaches and dizziness. An emergency CT scan revealed hemorrhage in the superior cerebellar cavity, ventricular, and subarachnoid compartments. Multiple cerebral hemorrhages in the patient were assumed to be the result of MMD. An urgent ventricular draining was done. The patient started experiencing disturbing consciousness on May 6. He underwent an urgent head CT scan, showed that increased intraventricular hemorrhage. On May 7, we carried out a DSA of the patient’s brain following the family’s informed consent. The angiographic results showed that the distal vessels of the right internal carotid artery were blurred, and severely stenosed. The right posterior communicating artery is provided for the majority of the anterior circulation. The shape and development of the right common carotid artery were normal. In the brain, the posterior cerebral arteries and superior cerebellar arteries are seen bilaterally. The basilar arteries were functioning and developing normally, and the posterior cerebral arteries compensate for part of the anterior circulation. The distal left internal carotid artery at the origin of the posterior communicating artery was significantly narrowed, the distal vessels showed smoky characteristics, and the saccular dilatation was observed at the distal end of the anterior choroidal artery. It was approximately 4.1*3.8 mm in size, which was considered a distal moyamoya aneurysm (Fig. 2 A–C). The lateral anterior choroidal artery could not be accessed during aneurysm embolization because it was too twisted, therefore the catheter had to be withdrawn. On May 7, the patient underwent a CTA of the brain to identify the position of the aneurysm. The results showed that Moyamoya disease progressed and the aneurysm was found on the left thalamus (paraventricular third ventricle) (Fig. 2 D and E).On May 8, the patient suddenly experienced epilepsy, the rigidity of the limbs, convulsions, vomiting, an increase in blood pressure to 200/105 mm Hg, and a dampened reflex to light. An emergency head CT revealed that the patient had more of a left basal ganglia brain hemorrhage that had entered the ventricular system (higher density shadow) (Fig. 2 F). After consulting with the patient’s family and getting their informed consent, an urgent intracerebroventricular hematoma evacuation and aneurysm excision were performed. To reach the left ventricle andresect aneurysm, the incision plan was designed (Fig. 3 A) and the corpus callosum was exposed once the dura was sliced open and some of the corpus callosum tissue was aspirated along with the interhemispheric fissure, and this all happened under a dissecting microscope. Bleeding was visible inside the third ventricle, and the interventricular foramen was enlarged. After the blood was evacuated, the third ventricle’s anterior and lateral walls were where the aneurysm was found (Fig. 3 B). An elongated parent artery is present. After the surrounding tissue was gently dissected, the aneurysm was found and resected.(Fig. 4 A).

On the first postoperative day, the patient showed up with around 4 fingers of neck stiffness, grade 1 extremity muscular strength on his right side. He developed lethargy and cognitive impairment, which were deemed to be signs of mutism, and might caused by corpus callosotomy, this neurological condition might go away on its own. On the second postoperative day, a head CT revealed a small amount of intraventricular bleeding, which was most noticeable in the 4th ventricle. Continuous drainage outside the ventricle and intermittent lumbar puncture released part of the cerebrospinal fluid. On the 4th postoperative day, a repeat DSA of the brain revealed that the original aneurysm had disappeared and no residual aneurysm was found (Fig. 4 B and C). On the 21st postoperative day, the patient’s GCS score returned to 15, and the left limb’s and right limb’s muscle strength were both grades of 5. The intraventricular hemorrhage was fully absorbed (Fig. 4 D). The patient was eventually released from the hospital after being able to walk with the help of family members.

## 3. Discussion

Although the exact cause of MMD is unknown, it is broadly accepted that genetic, immunological, and hemodynamic abnormalities play a role in both its initiation and progression.^[1]^ According to studies, there are several potential contributing factors to the complex etiology of moyamoya disease complicated by aneurysms, including genetics, inflammatory pathways, hemodynamic and vascular-related variables, and many others. The primary risk factor for the formation of cerebral aneurysms is genetics, and those with a family history are far more likely to develop MMD than the general population.^[8–11]^ Genetic factors are the greatest risk factors for developing intracranial aneurysms, and the incidence is significantly higher in people with a family history. Hirokazu Koseki et al^[12]^ claim that elevated wall shear stress (WSS), gradient oscillations, fluctuating hemodynamic pressures, mechanical tension, and high WSS all play a role in the development of cerebral aneurysms. WSS in particular is important in the development of aneurysms.^[10]^ Studies have connected the development and spread of brain aneurysms to inflammatory processes.^[13–15]^ Endothelial dysfunction has a considerable impact on aneurysm formation, and oxidative stress is regarded to be the main source of endothelial dysfunction.^[16]^ In addition to the abovementioned factors, the development of an aneurysm in this patient may be linked to an infection of the cerebrospinal fluid. Aortic and peripheral aneurysms make up the majority of complicated aneurysms in Moyamoya disease. The Willis ring is where the majority of aortic aneurysms develop.^[17–21]^ MMD with distal AChA aneurysms are very rare and the relevant literature does not provide a precise and effective treatment^[22,23]^

For patients with MMD complicated by aneurysm rupture and hemorrhage, interventional vascular embolization is advised because it is less invasive and patients recover quickly after surgery^[18,24–26]^; For the patients who do not have the conditions for interventional embolization, bypass revascularization, microscopic craniotomy or resection of aneurysms are better choices, and the patients can obtain a better prognosis.^[27]^

The 3 main surgical approaches to the anterior third ventricle include the frontal cortex-lateral ventricle-choroid fissure approach and the endplate approach as well as the interventricular foramen approach.^[28]^The frontal cortex-lateral ventricle-choroid fissure approach has a risk of postoperative seizures and may compromise cortical function. It is challenging to expose the lesion due to the lengthy endplate approach through the internal plate, which also runs the risk of harming the hypothalamus. The majority of the interventricular foramen approach through the interhemispheric corpus callosum is carried out in the area of brain tissue under the third ventricle, where there is minimal risk to neural tissue. It is important to note that the length of the corpus callosum incision should try not to exceed 2 cm. The location should not cross the coronal suture, otherwise the patient will have motor, intellectual, speech and other motor neuron dysfunction. Thus, in this report, we choose the interventricular foramen approach to the lateral corpus callosum and remove the aneurysm. Acha injury can lead to severe neurological deficits, so careful operation should be taken during operation.^[29]^ It was believed that the corpus callosum’s dissociation was responsible for mutism symptoms including mental lethargy, which may quickly improved on their own.^[30,31]^ The use of the CTA multimodal approach offered technical assistance for the identification of the aneurysm, reducing the difficulty of the surgery and potentially reducing operation time to some extent.

Depending on the circumstances, cerebral revascularization may be an alternative for patients who have MMD together with unruptured aneurysms.^[32,33]^ Direct, indirect, and mixed revascularization of the brain are all types of cerebral revascularization. An illustration is the bypass grafting of the superficial temporal artery-middle cerebral artery. In adults, it is difficult to create collateral circulation after indirect bypass grafting. Direct vascular bypass or combined procedures are recommended. Indirect revascularization mainly includes brain-muscle taping, brain-dura-arterial taping, brain-dura-arterial-temporal muscle taping, and intracranial multi-point drilling. Combined procedures can expand the scope of improving cerebral blood flow. Direct and combined revascularization, as opposed to indirect revascularization, can improve collateral circulation. In patients with MMD who have unruptured aneurysms and had a high risk of rupture, interventional embolization to remove risk factors is effective. According to certain studies, revascularization can be performed after a burst aneurysm’s bleeding has stabilized. To ascertain the effectiveness of this intervention, further study is required.

## 4. Conclusions

In conclusion, MMD with distal AChA aneurysms is extremely rare, and the pathophysiology is uncertain. Genetics, cerebral vascular hemodynamics, inflammation and immunology, and infections in the cerebrospinal fluid may all be involved. MMD with distal AChA aneurysms can be diagnosed and treated with the guidance of brain CTA or DSA, and removing the distal AChA aneurysms with craniotomy is an alternative that is generally successful and has a favorable prognosis.

## Acknowledgments

This work was supported by The Fundamental Research Funds for the Central Universities (2018KFYYXJJ105).

## Author contributions

**Data curation:** Lei Liu.

**Methodology:** Peng Wang, Wenchuan Qiu.

**Resources:** Hongwei Ren.

**Supervision:** Shengxi Jin.

**Writing – original draft:** Yunfei Zhao.

**Writing – review & editing:** Wei Ding.
